# Prognostic value of preoperative hematological markers combined with molecular pathology in patients with diffuse gliomas

**DOI:** 10.18632/aging.102186

**Published:** 2019-08-23

**Authors:** Zhen-Yu Zhang, Yun-Bo Zhan, Feng-Jiang Zhang, Bin Yu, Yu-Chen Ji, Jin-Qiao Zhou, Ya-Hui Bai, Yan-Min Wang, Li Wang, Yan Jing, Wen-Chao Duan, Chen Sun, Tao Sun, Hai-Biao Zhao, Ke Li, Wen-Qing Wang, Ruo-Yan Li, Hong-Wei Sun, Guang Zhai, Shu-Kai Wang, Xin-Ting Wei, Bo Yang, Dong-Ming Yan, Xian-Zhi Liu, Wei-Wei Wang

**Affiliations:** 1 Department of Neurosurgery, The First Affiliated Hospital of Zhengzhou University, Zhengzhou, Henan 450052, China; 2 Department of Pathology, The First Affiliated Hospital of Zhengzhou University, Zhengzhou, Henan 450052, China; 3 Department of MRI, The First Affiliated Hospital of Zhengzhou University, Zhengzhou, Henan 450052, China; 4 Department of SICU, The First Affiliated Hospital of Zhengzhou University, Zhengzhou, Henan 450052, China

**Keywords:** glioma, hematological marker, inflammation, molecular group, prognosis

## Abstract

The prediction of clinical outcome for patients with infiltrative gliomas is challenging. Although preoperative hematological markers have been proposed as predictors of survival in glioma and other cancers, systematic investigations that combine these data with other relevant clinical variables are needed to improve prognostic accuracy and patient outcomes. We investigated the prognostic value of preoperative hematological markers, alone and in combination with molecular pathology, for the survival of 592 patients with Grade II-IV diffuse gliomas. On univariate analysis, increased neutrophil-to-lymphocyte ratio (NLR), platelet-to-lymphocyte ratio (PLR), and monocyte-to-lymphocyte ratio (MLR), and decreased albumin-to-globulin ratio (AGR), all predicted poor prognosis in Grade II/III gliomas. Multivariate analysis incorporating tumor status based on the presence of *IDH* mutations, *TERT* promoter mutations, and 1p/19q codeletion showed that in lower-grade gliomas, high NLR predicted poorer survival for the triple-negative, IDH mutation only, TERT mutation only, and IDH and TERT mutation groups. NLR was an independent prognostic factor in Grade IV glioma. We therefore propose a prognostic model for diffuse gliomas based on the presence of *IDH* and *TERT* promoter mutations, 1p/19q codeletion, and NLR. This model classifies lower-grade gliomas into nine subgroups that can be combined into four main risk groups based on survival projections.

## INTRODUCTION

Gliomas are the most common malignant primary brain tumors, accounting for 27% of all central nervous system (CNS) tumors [1]. According to the World Health Organization (WHO) classification of CNS tumors, gliomas are pathologically categorized into four grades, of which Grade II to IV are considered diffusely infiltrating gliomas [2, 3].

Research on molecular alterations in gliomas has revealed three noteworthy biomarkers, namely codeletion of chromosome arms 1p and 19q (1p/19q codeletion), and mutations in *IDH* and the *TERT* promoter, that can be used to classify Grade II-IV gliomas into five principal molecular groups (triple-positive, *IDH* and *TERT* mutations, *IDH* mutation only, triple-negative, and *TERT* mutation only). These groups are associated with distinct prognosis, germline variants, and median age at diagnosis, highlighting different pathogenic mechanisms [3]. Although most glioma patients receive standard treatments, significant variations in clinical outcomes are often seen due to the heterogeneity of the tumors [4]. Therefore, it is necessary to identify more appropriate and effective biomarkers for predicting prognosis in glioma patients.

Inflammation and immunity are critically involved in glioma initiation and progression [5, 6], and several studies demonstrated that inflammatory response cells such as neutrophils [7], lymphocytes [8] and platelets [9] are associated with the prognosis of cancer patients. In recent years, the prognostic value of preoperative hematological markers, such as neutrophil-to-lymphocyte ratio (NLR), platelet-to-lymphocyte ratio (PLR), monocyte-to-lymphocyte ratio (MLR), median platelet volume (MPV), platelet distribution width (PDW), and albumin-to-globulin ratio (AGR), has been investigated in several cancers, including gliomas [10–17]. However, there is a lack of studies systematically investigating the prognostic value of hematological markers in a large cohort of gliomas, particularly in relation to the different molecular subtypes.

Therefore, we investigated the prognostic value of preoperative hematological markers (NLR, PLR, MLR, MPV, PDW, and AGR), alone and in combination with the 5 glioma molecular groups, on the clinical outcomes of a relatively large cohort (n = 592) of Grade II-IV glioma patients. Based on these findings, we propose a prognostic model for Grade II-IV infiltrative gliomas based on molecular pathology and NLR, and identify for lower-grade (WHO Grade II and III) gliomas, four risk groups with distinct overall survival. Further validation of the model in more extensive cohorts should confirm its usefulness and possibly open the way to new therapeutic strategies.

## RESULTS

### Clinico-pathological characteristics of the cohort

A total of 592 cases (adult patients, age ≥ 16) of WHO Grade II-IV supratentorial gliomas were analyzed. The median age of the cohort was 42 years (interquartile range = 39–58 years). There were 335 male patients (56.6%) and 257 female patients (43.4%). The cohort included 404 patients (68.2%) with Grade II-III glioma and 188 patients (31.8%) with Grade IV glioma. Median duration of follow-up was 32.0 months. Complete resection was achieved in 456 patients (77%), and incomplete resection was performed in 136 patients (23%). Four hundred and fifty-nine patients (77.5%) received postoperative primary radiation therapy (RT) and 342 patients (57.8%) received postoperative primary chemotherapy (CHT). In patients with astrocytoma, 14 (9.0%) received postoperative primary RT, 10 (6.5%) received postoperative primary CHT, 113 (72.9%) received postoperative primary RT and CHT, and 18 (11.6%) received no postoperative treatment. Among patients with oligodendroglioma or oligoastrocytomas, 31 (12.5%) received postoperative primary RT, 24 (9.7%) received postoperative primary CHT, 166 (66.9%) received postoperative primary RT and CHT, and 27 (10.9%) received no postoperative treatment (Supplementary Table 3). Molecular pathology analyses were available for 573 cases. *IDH* mutations were found in 246 cases (42.9%), mutations in *TERT* promoter were detected in 286 cases (49.9%), and chromosome 1p/19q codeletion was detected in 139 cases (34.4%). Hematological markers were defined in 528/592 cases, as 64 cases were excluded due to conditions that could influence peripheral blood counts. Detailed information on the clinico-pathological features of the cohort is listed in Supplementary Table 1.

### Molecular groups

**Figure 1:**
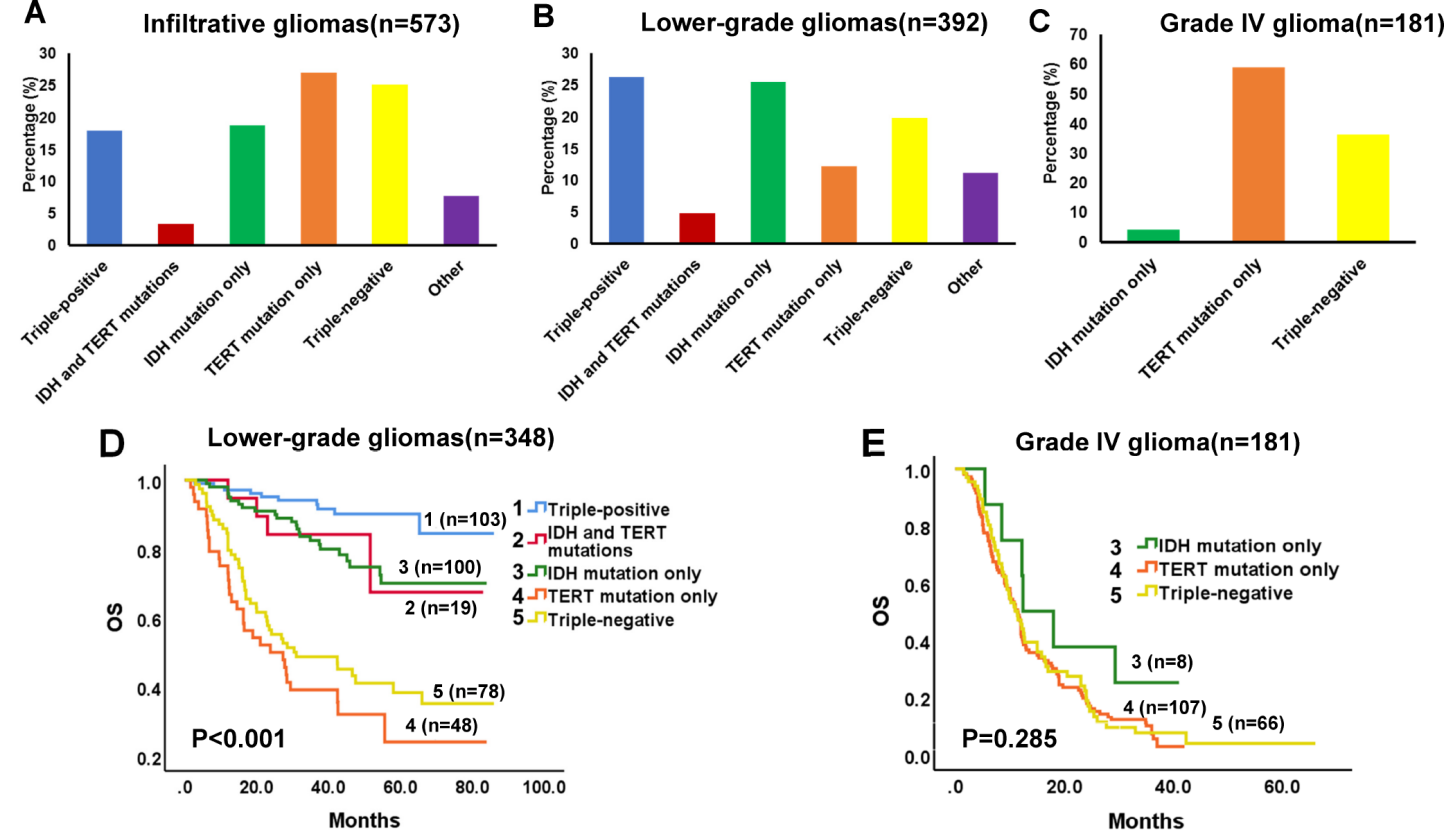
**Proportion and Kaplan-Meier survival analyses of molecular groups in diffuse infiltrative gliomas.** Survival proportions in infiltrative (Grade II-IV) gliomas (**A**), lower-grade (Grade II-III) gliomas (**B**), and Grade IV glioma (**C**). (**D**) Kaplan-Meier OS curves in lower-grade gliomas. OS estimates for the 5 molecular groups are significantly different (P < 0.001). (**E**) Kaplan-Meier OS curves in Grade IV glioma. No differences in OS were detected for the 3 molecular groups (P = 0.285).

Among the 573 cases of Grade II-IV gliomas, 103 (18.0%) were triple-positive, 19 (3.3%) had mutations in both *IDH* and *TERT*, 108 (18.8%) had *IDH* mutation only, 144 (25.1%) were triple-negative, 155 (27.1%) had *TERT* mutation only, and 44 (7.7%) had other combinations of the three biomarkers (Figure 1A). For lower-grade glioma cases (n = 392), 103 (26.3%) were triple-positive, 19 (4.8%) had both *IDH* and *TERT* mutations, 100 (25.5%) had *IDH* mutation only, 48 (12.24%) had *TERT* mutation only, 78 (19.9%) were triple-negative, and 44 (11.2%) had other combinations (Figure 1B). For Grade IV glioma cases (n = 181), 8 (4.4%) had *IDH* mutation only, 107 (59.1%) had *TERT* mutation only, and 66 (36.5%) were triple-negative (Figure 1C). Univariate survival analysis demonstrated that molecular groups significantly influenced the OS of patients with lower-grade gliomas. The triple-positive group had favorable prognosis, whereas the *TERT* mutation group had a dismal survival expectancy (Figure 1D, univariate analysis in Supplementary Table 2), although this relationship was not found for Grade IV gliomas (Figure 1E, Supplementary Table 5). In subsequent multivariate analysis, molecular group was revealed as an independent prognostic factor in lower-grade gliomas (Table 1).

**Table 1:** Multivariate analysis of adjusting putative prognostic factors for molecular group (n=348^a^) and risk group (n=348^a^) in lower-grade gliomas.

**Factors**	**OS**	
	**HR (95%CI)**	**P-value**
Molecular group	1.578 (1.366–1.822)	**<0.001**
Age	1.883 (1.220–2.908)	**0.004**
Extent of resection	1.262 (0.813–1.960)	0.300
RT (Yes or No)	1.770 (1.147–2.732)	**0.010**
Grade (II or III)	3.408 (2.300–5.049)	**<0.001**
KPS (≤80 or >80)	0.693 (0.478–1.006)	0.054
		
Risk group	1.214 (1.149–1.283)	**<0.001**
Age (≤40 or >40)	1.630 (1.061–2.504)	**0.026**
Extent of resection	1.122 (0.721–1.745)	0.610
RT (Yes or No)	1.772 (1.153–2.724)	**0.009**
KPS (≤80 or >80)	0.742 (0.510–1.079)	0.118
Grade (II or III)	3.112 (2.091–4.634)	**<0.001**

^a^12 cases were excluded due to unavailability of FFPE tissues of the tumors, and 44 cases of other combinations of the three molecular markers were excluded.

Molecular group: the five molecular groups based on the statuses of *IDH* mutations, *TERT* promoter mutations and 1p/19q co-deletion, which include triple positive, *IDH* and *TERT* mutations, *IDH* mutation only, *TERT* mutation only and triple negative).

Risk group: the four groups based on the statuses of *IDH* mutations, *TERT* promoter mutations, 1p/19q codeletion and the levels of NLR in each molecular subgroup

OS: overall survival

RT: radiation therapy, indicating postoperative radiation therapy after first operation

KPS: Karnofsky Performance Status

HR: Hazard-ratio

### Prognostic value of hematological markers in lower-grade and Grade IV gliomas

**Figure 2:**
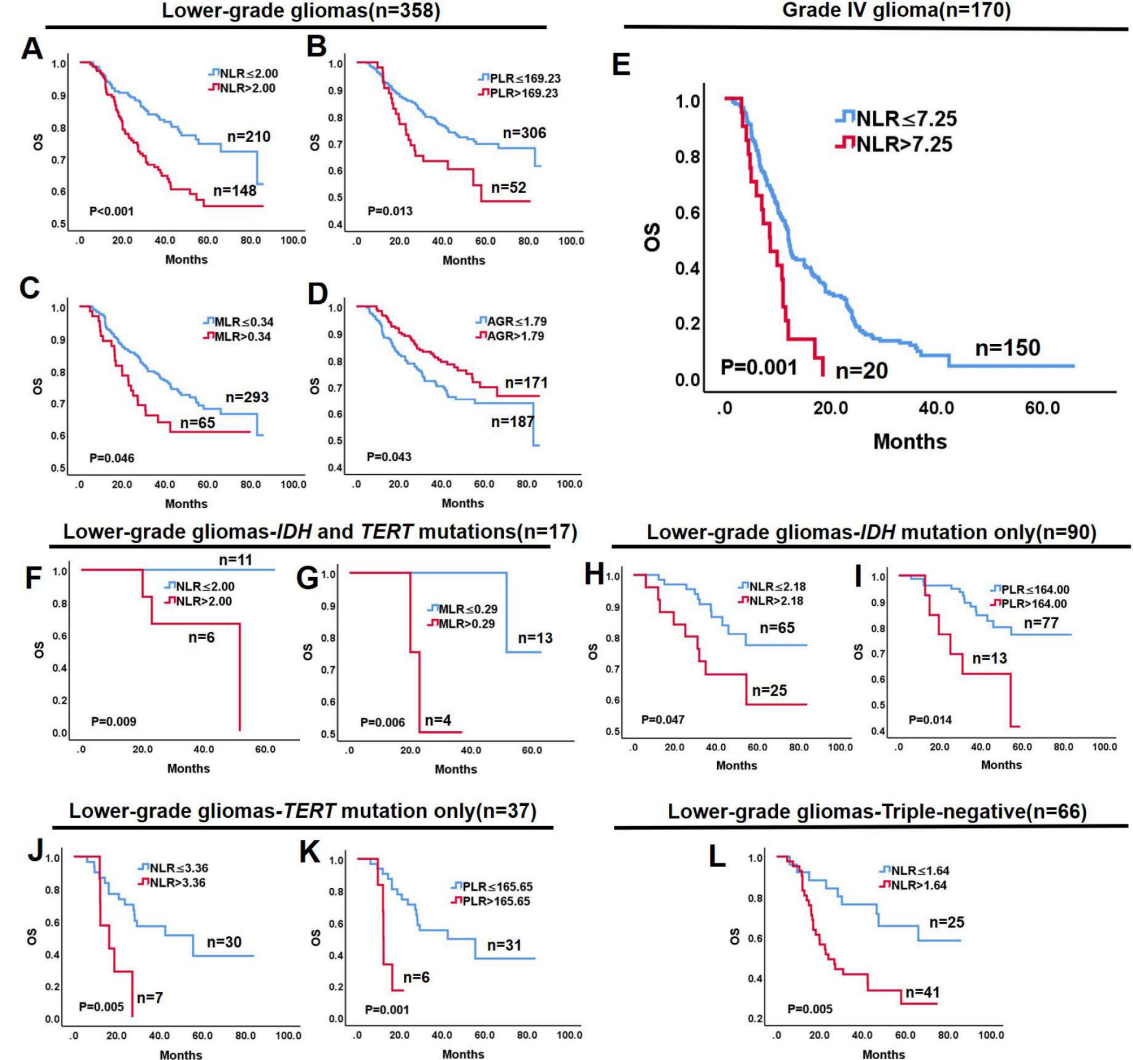
**Kaplan-Meier survival curves of glioma subgroups based on hematological markers.** Lower-grade gliomas: (**A**) NLR > 2.00 is associated with worse OS (P < 0.001); (**B**) PLR > 169.23 is associated with worse OS (P = 0.013); (**C**) MLR > 0.34 is associated with worse OS (P = 0.046); and (**D**) AGR > 1.79 is associated with better OS (P = 0.043). (**E**) In Grade IV glioma, NLR > 7.25 is associated with better OS (P = 0.001). (**F**, **G**) In the IDH and TERT mutations group of lower-grade gliomas, NLR > 2.00 and MLR > 0.29 predict worse OS (P < 0.009 and P = 0.006, respectively). (**H**, **I**) In the IDH mutation only group of lower-grade gliomas, NLR > 2.18 and PLR > 164.00 predict worse OS (P = 0.047 and P = 0.014, respectively). (**J**, **K**) In the TERT mutation only group of lower-grade gliomas, NLR > 3.36 and PLR > 165.65 predict worse OS (P = 0.005 and P = 0.001, respectively). (**L**) In triple-negative lower-grade gliomas, NLR > 1.64 predicts worse OS.

The prognostic value of the hematological markers was evaluated in lower-grade gliomas and Grade IV gliomas. Optimal cut-off values of NLR, PLR, MLR, MPV, PDW, and AGR were computed by X-tile software. Univariate analysis demonstrated that high NLR (P < 0.001), PLR (P = 0.013), and MLR (P = 0.046), and low AGR (P = 0.043) were associated with shorter survival in lower-grade gliomas (Figure 2A–2D), while MPV (P = 0.204) and PDW (P = 0.422) had no prognostic significance. Because hematological markers were strongly correlated and interfered with each other [14], they were separately analyzed with other potential prognostic factors in multivariate analysis. The latter revealed that NLR (P = 0.046, Table 2) is a prognostic factor for lower-grade gliomas independent of age, extent of resection, and adjuvant therapies. Conversely, neither PLR (P = 0.102), MLR (P = 0.188), nor AGR (P = 0.621) were independent prognostic factors (Supplementary Table 4). In Grade IV gliomas, only NLR emerged as a significant prognostic factor in univariate (P = 0.001, Figure 2E) and multivariate (P = 0.002, Table 3) analyses.

**Table 2:** Multivariate analysis of adjusting putative prognostic factors for NLR (n=358^a^) in lower-grade gliomas.

**Factors**	**OS**	
	**HR (95%CI)**	**P-value**
NLR	1.502 (1.007–2.240)	**0.046**
Age	1.042 (1.024–1.060)	**<0.001**
Extent of resection	0.907 (0.540–1.524)	0.713
RT (Yes or No)	0.860 (0.514–1.440)	0.567
Grade (II or III)	3.746 (2.499–5.618)	**<0.001**
KPS (≤80 or >80)	0.618 (0.416–0.916)	**0.017**

^a^46 cases were excluded due to conditions that could influence hematological makers

OS: overall survival

RT: radiation therapy, indicating postoperative radiation therapy after first operation

KPS: Karnofsky Performance Status

HR: Hazard-ratio

**Table 3:** Multivariate analysis of adjusting putative prognostic factors for NLR in Grade IV glioma (n=170^a^).

**Factors**	**OS**	
	**HR (95%CI)**	**P-value**
NLR	2.228 (1.329–3.733)	**0.002**
Extent of resection	2.815 (1.952–4.059)	**<0.001**
RT (Yes or No)	1.213 (0.772–1.907)	0.402
CHT (Yes or No)	1.339 (0.871–2.061)	0.184
Age (≤62 or >62)	1.587(1.103–2.284)	**0.013**

^a^18 cases were excluded due to conditions that could influence hematological makers

OS: overall survival

RT: radiation therapy, indicating postoperative radiation therapy after first operation

CHT: chemotherapy, indicating postoperative chemotherapy after first operation

HR: Hazard-ratio

### Prognostic value of hematological markers within molecular groups of lower-grade gliomas

Since molecular subtype is an independent factor influencing survival in lower-grade gliomas but not Grade IV glioma, we evaluated the prognostic value of hematological markers for each molecular group in lower-grade gliomas. Optimal cut-off values for NLR, PLR, MLR, and AGR in each group were computed by X-tile software. As shown in Figure 2F–2L, univariate analysis showed that high NLR predicted shorter OS in lower-grade glioma groups defined by *IDH* and *TERT* mutations (P = 0.009), *IDH* mutation only (P = 0.047), *TERT* mutation only (P = 0.005), and in the triple-negative group (P = 0.005). In turn, high PLR predicted shorter OS in the *IDH* mutation only (P = 0.014) and *TERT* mutation only (P = 0.001) groups, while high MLR was associated with shorter OS in gliomas with *IDH* and *TERT* mutations (P = 0.006). In contrast, none of the hematological markers impacted OS in the triple-positive group of lower-grade gliomas (Supplementary Figure 1A–1D). Likewise, no prognostic significance was found for PLR and AGR in the *IDH* and *TERT* mutation group (Supplementary Figure 1E, 1F), MLR and AGR in the *IDH* mutation only group (Supplementary Figure 1G, 1H), MLR and AGR in the *TERT* mutation only group (Supplementary Figure 2A, 2B), and PLR, MLR, and AGR in the triple-negative group (Supplementary Figure 2C–2E) of lower-grade gliomas.

### A glioma prognostic model combining molecular pathology and hematological markers

**Figure 3:**
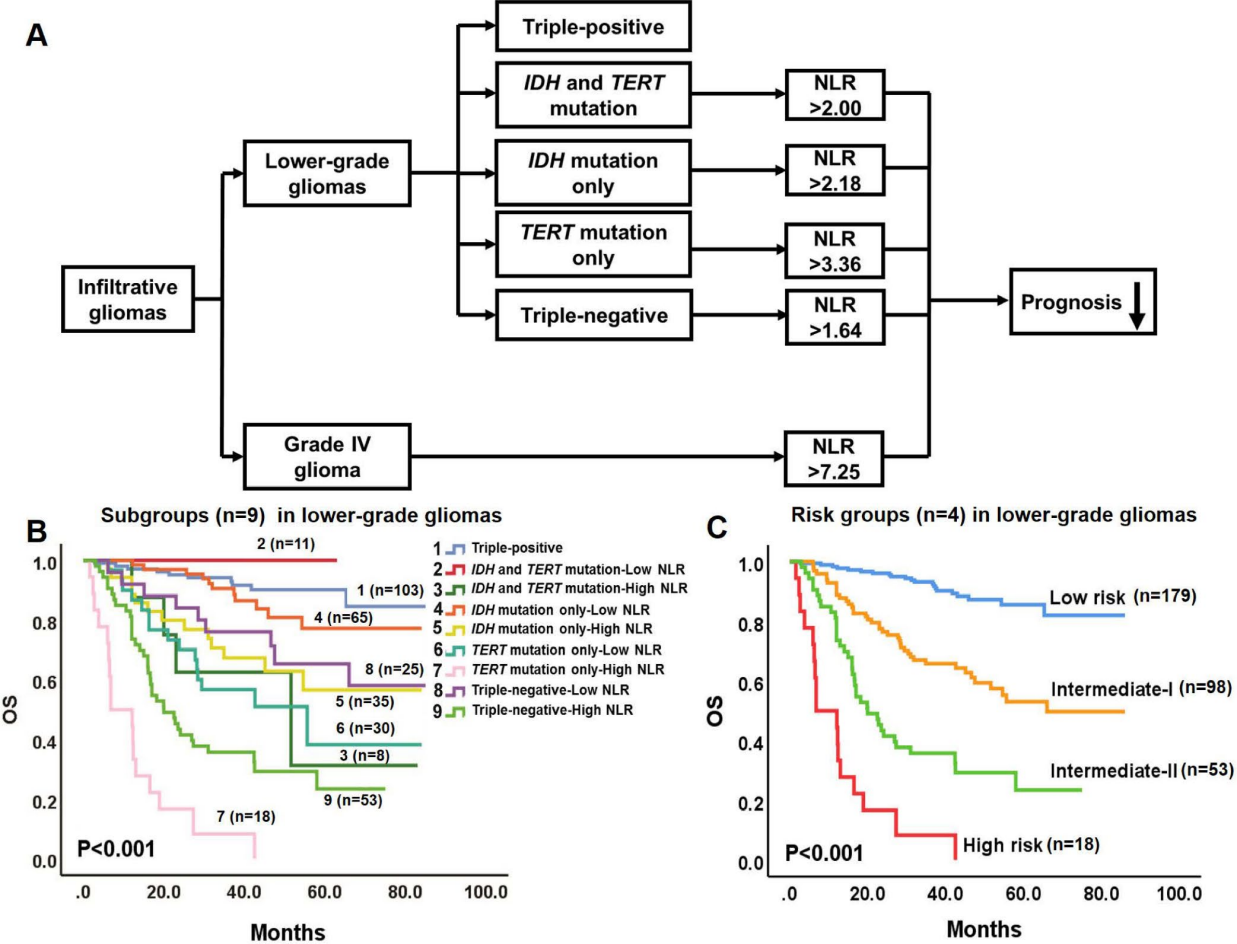
**Prognostic model combining molecular pathology and hematological markers for gliomas.** (**A**) In the model, infiltrative gliomas include lower-grade gliomas and Grade IV glioma. Lower-grade gliomas are divided into 5 molecular groups with different OS. NLR is proposed to further stratify the OS of 4 of these groups (triple-positive tumors are excepted). In Grade IV glioma, high NLR independently predicts worse OS. (**B**) Kaplan-Meier OS curves of the 9 subgroups of lower-grade gliomas. The OS of the 9 subgroups are significantly different (P < 0.001). (**C**) Kaplan-Meier OS curves of the 4 risk groups in lower-grade gliomas: Subgroups 1, 2, and 4 were integrated into a Low risk group; Subgroups 3, 5, 6, and 8 were integrated into an Intermediate-I risk group; Subgroup 9 comprises the Intermediate-II risk group; and Subgroup 7 constitutes the High risk group. The OS of the 4 risk groups differs significantly (P < 0.001).

Based on combined data derived from survival analyses of molecular pathology and hematological markers, we propose a prognostic model to predict survival in glioma patients (Figure 3A). In the model, infiltrative gliomas include lower-grade gliomas and Grade IV glioma. Lower-grade gliomas were divided into 5 primary molecular groups associated with distinct OS [3]. Since NLR is a prognostic factor independent of putative clinical variables in lower-grade gliomas, and predicts survival in 4 tumor subtypes (triple-negative, *IDH* and *TERT* mutations, *IDH* mutation only, and *TERT* mutation only), this hematological marker is proposed to further stratify the prognosis of these 4 molecular groups. In contrast, high NLR arises as an independent predictor of worse survival for Grade IV glioma.

### Molecular pathology and NLR stratify lower-grade gliomas into four risk groups

According to the prognostic model proposed in Figure 3A, lower-grade gliomas were categorized into nine subgroups based on the status of *IDH* and *TERT* promoter mutations, 1p/19q codeletion, and NLR. Survival analyses revealed significantly different OS for these nine subgroups (P < 0.001, Figure 3B, Supplementary Table 6). Furthermore, subgroups with non-significant differences in OS between them were integrated into individual risk groups: Subgroups 1, 2, and 4 conformed the Low risk group, Subgroups 3, 5, 6, and 8 conformed the Intermediate-I risk group, Subgroup 9 was defined as the Intermediate-II risk group, and Subgroup 7 represented the High risk group (Figure 3B, 3C). Univariate (P < 0.001, Figure 3C, Supplementary Table 7) and multivariate (P < 0.001, Table 1) analyses yielded significantly different OS for these four risk groups.

## DISCUSSION

In the present study, data from a large cohort of gliomas (n = 592) were used to corroborate previous findings on the 5 glioma molecular groups defined by three robust markers, 1p/19q codeletion, *IDH* mutations, and *TERT* promoter mutations [2] and to demonstrate, for lower-grade gliomas, the differential prognostic value of hematological markers in each molecular group. Based on these findings, we propose a prognostic model for infiltrative gliomas that combines molecular and hematological markers.

The involvement of tumor-associated inflammatory cells in carcinogenesis has been firmly established [5, 7]. Cancer cells secrete chemokines and cytokines that attract host inflammatory cells such as neutrophils and lymphocytes, and these cells produce in turn proinflammatory cytokines, growth factors, and chemokines that contribute to tumor progression [18–20]. Unlike genetic biomarkers, preoperative hematological markers can be easily calculated from routine blood tests and may have important clinical significance for cancer prognosis. In recent years the prognostic value of NLR, PLR, MLR, and AGR has been investigated and corroborated in several cancers, such as hepatocellular carcinoma [21], pancreatic carcinoma [22], renal carcinoma [11], esophageal cancer [23], gastric carcinoma [24], colorectal cancer [25], lung cancer [26] and gliomas [14–17, 27, 28].

Our study demonstrated that NLR, PLR, MLR, and AGR are prognostic factors in univariate analysis for lower-grade gliomas. Also, for these tumors, multivariate analysis revealed that NLR is an independent prognostic factor after adjusting for age, grade, histology, extent of resection, and adjuvant therapies.

Studies demonstrated that neutrophil-induced immunosuppression can promote glioma progression, and that certain subsets of T-lymphocytes can instead inhibit it via induction of cytotoxic cell death and cytokine production [20, 29, 30]. Accordingly, Han et al. reported that high neutrophil and low CD3+ T-cell infiltration (elevated NLR) in glioblastomas was correlated with poorer outcomes [15]. Evidence for the importance of PLR in oncogenesis comes from studies showing that platelet activation contributes to tumor angiogenesis, disruption of the extracellular matrix and release of adhesion molecules to promote cancer cell proliferation and metastasis [31, 32]. As for AGR, its relevance in cancer may be related to the antioxidative effects of albumin against carcinogens such as nitrosamines and aflatoxins [33], and the association of elevated globulin levels with the progression and metastasis of some cancers [34].

Although predicting the clinical outcome of infiltrative gliomas is challenging, considerable progress in the classification of gliomas based on molecular markers has been made in the past several years [2, 3, 35–39]. Particularly, three robust molecular alterations, namely 1p/19q codeletion and *IDH* and *TERT* promoter mutations, were used to categorize five principal molecular groups of gliomas with distinct clinical traits and mechanisms of carcinogenesis [2]. Chromosome 1p/19q codeletion is associated with oligodendrogliomas, sensitivity to adjuvant therapies, and favorable survival [35, 40]. Mutations in *IDH* genes (*IDH*1 and *IDH*2) have been revealed in the majority of lower-grade gliomas and in secondary glioblastoma multiforme, and predict better survival [36, 41]. In a previous study we demonstrated that *TERT* promoter mutations could identify among lower-grade gliomas a group of *IDH*-mutated-1p/19q-intact tumors with better survival and a subset of *IDH* wild-type tumors with worse prognosis [42]. At present, the classification of infiltrative gliomas based on these three molecular markers is routinely conducted and of vital significance in clinical practice [3]. Based on this scheme, through multivariate survival analysis on 573 adult infiltrative gliomas we confirmed in lower-grade tumors the prognostic significance of the principal molecular groups independent of age, histology, and clinical variables. Our results further corroborate the findings reported by Eckel-Passow et al. [3] while adding several key clinical variables omitted in their research. Meanwhile, consistent with Eckel-Passow et al., for WHO Grade IV gliomas the molecular groups lacked independent prognostic significance.

We investigated for the first time, to the best of our knowledge, the prognostic value of hematological markers within the 5 primary glioma molecular groups and found that for lower-grade gliomas, high NLR and MLR predicted worse survival in the *IDH* and *TERT* mutations group, high NLR and PLR predicted worse survival in the *IDH* mutation only and *TERT* mutation only groups, and high NLR was associated with shorter survival in the triple-negative group. Interestingly, no predictive value was found for any hematological marker in triple-positive tumors. We speculate that any potential contribution to prognosis may be masked by the favorable survival characteristic of lower-grade gliomas within this molecular group. The differential prognostic values found for these hematological markers may be related to distinct immune microenvironments associated with specific molecular groups. For example, Qian et al. reported that immune responses in lower-grade gliomas are regulated by *IDH* mutations [43]. In Grade IV glioma, NLR was revealed as an independent prognostic factor in multivariate analysis, while the predictive values of PLR, MLR, and AGR were not significant in univariate analyses. We therefore developed a prognostic model for infiltrative gliomas by combining molecular and hematological markers. The model identified four risk groups based on molecular pathology and NLR in lower-grade gliomas, and two risk groups based on NLR in Grade IV glioma. The model only requires information of routine preoperative blood tests and molecular analysis of 1p/19q codeletion and *IDH* and *TERT* promoter mutations, which is also available in most medical centers. We think the model can be used readily and easily in the clinic, after corroboration from a multi-center, prospective clinical trial.

The present study has some limitations. First, due to the retrospective nature of the study, systematic bias might influence the accuracy of the results. Second, although the current study enrolled a relatively large sample size, it was carried out in a single research center. Thus, multi-center, prospective studies are necessary to corroborate our findings. Lastly, more extensive research is needed to clarify the detailed mechanisms through which hematological markers influence the prognosis of molecular groups in gliomas.

In summary, our study corroborates the prognostic significance of glioma subtypes based on 1p/19q codeletion and *IDH* and *TERT* promoter mutations in a large Chinese cohort. Moreover, we propose a novel prognostic model for diffuse infiltrative gliomas that combines molecular pathology and hematological markers, and may increase prognostic accuracy and improve patient outcomes.

## MATERIALS AND METHODS

### Study cohort

This study was approved by the Human Scientific Ethics Committee of the First Affiliated Hospital of Zhengzhou University. Five hundred and ninety-two cases of infiltrative gliomas (WHO II, III and IV) with complete follow-up data were enrolled in the study. Patients in the cohort were surgically treated in the First Affiliated Hospital of Zhengzhou University from 2011 to 2016. The diagnosis was made by pathological examination and centrally reviewed by two pathologists according to the 2016 WHO classification of tumors of the CNS [2]. All patients enrolled in the current study were treatment-naïve (i.e neither surgical resection, chemotherapy, nor radiotherapy were administered before the first operation). For survival analysis of hematological markers, patients with hematological diseases, serious infections, surgery, trauma, and anti-coagulant therapy were excluded. All clinical data, including gender, age, preoperative Karnofsky Performance Status (KPS) score, extent of resection, histological grade, and adjuvant therapies were collected from the medical record system. Follow-up data were acquired by telephone or out-patient follow-up. Overall survival (OS) was calculated as the time interval between the date of surgery and the date of death or the end of follow-up.

### Molecular classification

Formalin-fixed, paraffin embedded (FFPE) tissues were available in 573 cases. The detection of molecular markers was centrally conducted with standardized protocols. Mutational hotspots in *IDH*1, *IDH*2, and the *TERT* promoter were detected by Sanger sequencing. Chromosome 1p/19q status was evaluated by fluorescence in situ hybridization in all WHO Grade II and Grade III gliomas. Detailed protocols are described in the Supplementary Materials. According to the status of the three molecular markers, infiltrative gliomas were categorized into five principal groups: triple-positive (mutations in *TERT* promoter and *IDH*, plus 1p/19q codeletion); mutations in both *TERT* and *IDH*; mutation in *IDH* only; mutation in *TERT* only; and triple- negative [3].

### Hematological markers

Routine preoperative blood and hepatic function tests prior to the first surgical resection were centrally performed at the Department of Clinical Laboratory within 2 hours of blood sample collection. Blood test results included neutrophil, lymphocyte, mononuclear cell, and platelet counts, as well as mean platelet volume and platelet distribution width. Results of the hepatic function test included albumin and globulin levels to calculate AGR. Hematological markers included: NLR = neutrophil-to-lymphocyte ratio, PLR = platelet-to-lymphocyte ratio, MLR = monocyte-to- lymphocyte ratio, MPV = median platelet volume, PDW = platelet distribution width, and AGR = albumin-to-globulin ratio.

### Statistical methods

SPSS 19.0 (IBM Corp., Armonk, NY, USA), Graph-Pad Prism 6.0 (Graph-pad Inc, La Jolla, USA) and X-tile 3.6.1 (http://medicine.yale.edu/lab/rimm/research/ software.aspx) were used to analyze the data. The Kaplan-Meier method and the log-rank test were used to calculate survival rates. Post-hoc Bonferroni test was used for multiple comparisons. Multivariate analysis using Cox regression was performed to evaluate independent prognostic factors. P < 0.05 was considered statistically significant.

## Supplementary Material

Supplementary Methods

Supplementary Figures

Supplementary Tables
