# Supplementary material for: Prognostic value of preoperative hematological markers combined with molecular pathology in patients with diffuse gliomas
Source: Aging (Albany NY). 2019 Aug 23;11(16):6252–72. doi: 10.18632/aging.102186 (PMC6738441; doi:10.18632/aging.102186)
Supplement: Supplementary Methods [file aging-11-102186-s001.pdf]

SUPPLEMENTARY METHODS

Mutation analysis of *IDH*1/*IDH*2

Mutational hotspots of *IDH1/IDH2* were evaluated by direct sequencing. Tissues from representative tumor area (the proportion of tumor cells>20%) were scrapped off from dewaxed sections and treated with PCR reaction solution A 10μl (reaction mixture containing 1μl of cell lysate, 0.3mM of each dNTP, 2.5mM $MgCl_2$, 0.3μM of each primer and 0.2U of KAPA HiFi HotStart DNA Polymerase (Kapa Biosystems Inc., Wilmington, USA)), Shrimp Alkaline Phosphatase (SAP) enzyme (NEB, Ipswich, MA, USA) 2μl and BigDye (BigDye™ Terminator v3.1 Cycle Sequencing Kit, Thermo Fisher Scientific, Waltham, MA, USA) 1μl for centrifugation at 2000 rpm for 10 sec. The crude cell lysate was centrifuged and supernatant was used for subsequent PCR analysis. The forward primer primers (*IDH*1-F:5'-CGGTCTTCAGAGAAGCCATT-3',*IDH*1-R:5'-CACAT TATTGCCAACATGAC-3',*IDH*2-F:5'-AGCCCATCAT CTGCAAAAAC-3',*IDH*2-R:5'-CTAGGCGAGGAGCT CCAGT-3') were used to amplify the region of mutational hotspots of *IDH1/IDH2*. [illegible]PCR was performed was initiated at 95°C for 5 min, followed by 40 cycles of 95°C for 20 sec, 57°C for 30 sec and 72°C for 1min, and a final extension of 72°C for 5 min and 10°C for 10 min. [illegible]5μl PCR products were then mixed with 2μl SAP enzyme and reacted at 37°C for 40min and then at 80° C for 15min. [illegible] Then 18μl PCR reaction solution C (CWBIO, Beijing, Chima), 1μl products from [illegible] step, and 1μl BigDye were mixed and reacted at 96°C for 1 min, followed by 30 cycles of 96°C for 10 sec, 50° C for 5 sec and 60° C for 2 min, and a final extension of 25°C for 1 min and 10°C for 10 min. Then 50μl natrium asceticism-ethanol mixture (3M NaAc: ethanol=1:15) were added and the mixture was centrifuged for 30min (12000 rpm, 4°C), with the supernatant being discarded. Then 70μl 75% ethanol were added and the mixture was centrifugated for 15min (12000 rpm, 4°C), with the supernatant being discarded. After complete volatilization of the ethanol at room temperature, 12μl Hi-Di™ Formamide (Thermo Fisher Scientific, Waltham, MA, USA) were added into the precipitate to dissolve the DNA. The dissolved products were sequenced on Applied Biosystems™ 3500DxGenetic Analyzer (Thermo Fisher Scientific, Waltham, MA, USA), and analyzed by Chromas software (Technelysium, South Brisbane, Australia). The sequencing results were compared with wild-type sequences of IDH1/IDH2 for analysis.

Mutation analysis of *TERT* promoter

Tissues sample were prepared according to the "Mutation Analysis of *IDH*1/*IDH*2 protocol" protocol previous described. The crude cell lysate was centrifuged and supernatant was used for subsequent PCR analysis. The forward primer *TERT*-F (5'-GTCCTGCCCCTTCACC TT-3') and reverse primer *TERT*-R (5'-CAGCGCTGCC TGAAACTC-3') were used to amplify a 163bp fragment spanning the two mutational hotspots [chr5, 1,295,228 (C228T) and 1,295,250 (C250T)] in *TERT* promoter region. [illegible]PCR was performed was initiated at 95°C for 5 min, followed by 40 cycles of 95°C for 20 sec, 57°C for 30 sec and 72°C for 1min, and a final extension of 72°C for 5 min and 10°C for 10 min. [illegible]5μl PCR products were then mixed with SAP enzyme and reacted at 37°C for 40 min and then at 80° C for 15 min. [illegible]Then 18μl PCR reaction solution C, 1μl products from [illegible] step, and 1μl BigDye were mixed and reacted at 96°C for 1 min, followed by 30 cycles of 96°C for 10 sec, 50° C for 5 sec and 60° C for 2min, and a final extension of 25°C for 1 min and 10°C for 10 min. The following steps were performed according to the "Mutation Analysis of *IDH*1/*IDH*2 protocol" protocol previous described. The sequencing results were compared with wild-type sequences of TERT for analysis.

Chromosome 1p/19q status by Fluorescence in Situ Hybridization (FISH)

Chromosome 1p/19q status was examined by fluorescence in situ hybridization. 4μm thick FFPE sections were baked at 65°C for 2-3h and deparaffinized in xylene for 10 minutes for 2 times. The sections were hydrated by 100% ethanol for 2 min, 85% ethanol for 2 min and 70% ethanol for 2 min orderly, and then immerged in deionized water for 3 min. The sections were processed with citrate repair solution in (pH6.0) for 4 min in high pressure condition, and then rinsed in 2×SSC solution for 5 min for 2 times. The sections were immerged in protease K fluid (200μg/ml) and incubated for 2 min at 37°C, and then rinsed in 2×SSC solution for 5 min for 2 times. 10μl probes (GP Medical Technologies, Beijing, China) mixture was added to the hybridization zone of the section, and the denaturation and hybridization process was carried out on the ThermoBrite®hybridization instrument (Leica Biosystems, Nussloch, Germany), with denaturation temperature at 83°C for 5 min and hybridization temperature at 42°C for 16h. Sections were immerged in 0.4×SSC plus 0.3% NP-40 cleaning solution (65±1°C) and vibrated for 3 sec. Sections were then retrieved 2 min later and put into 0.1% NP-40 plus 2×SSC cleaning solution at room temperature, vibrated for 3 sec and cleansed for 1 min. Then the sections were immerged in 70% ethanol for 3 min and dried avoiding light at room temperature. 15μl DAPI redyeing agent was added into the hybridization zone of the section, and the section was placed avoiding light for 10 min. At last, the section was placed under the BX51TRF

fluorescence microscope (Olympus, Tokyo, Japan) for analysis by expert pathologist (Dr. Wei-wei Wang). Hybridizing signals in at least 100 non-overlapping nuclei were counted. The loci interrogated were 1p36.3 (RP11-62M23 labeled red)/1q25.3-q31.1 (RP11-162L13 labeled green) and 19q13.3 (CTD-2571L23 labeled red)/19p12 (RP11-420K14 labeled green). A sample was considered 1p or 19q deleted according to the ratio of number of red signal to green signal. In 1p36 or 19q13, positive loss of heterozygosity (LOH) was determined when the ratio of number of red signal to green signal was less than 0.7.
