# Supplementary material for: Prognostic value of preoperative hematological markers combined with molecular pathology in patients with diffuse gliomas
Source: Aging (Albany NY). 2019 Aug 23;11(16):6252–72. doi: 10.18632/aging.102186 (PMC6738441; doi:10.18632/aging.102186)
Supplement: Supplementary Figures [file aging-11-102186-s003.pdf]

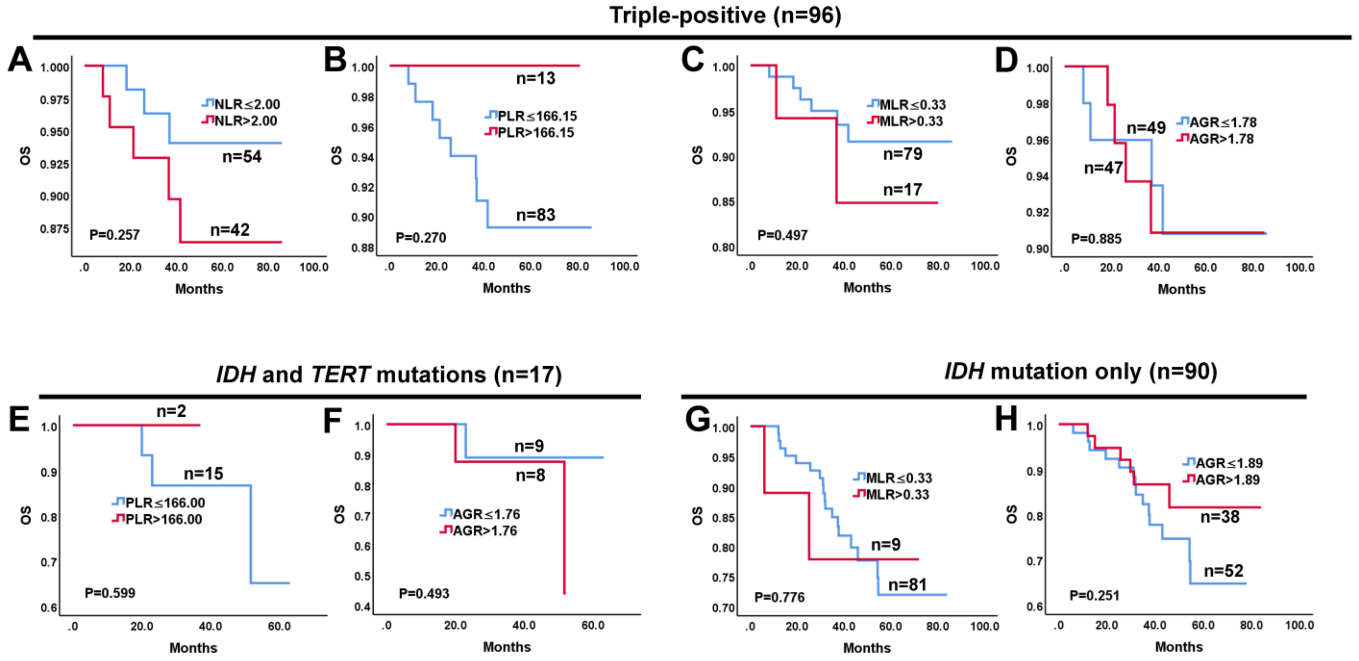

Supplementary Figure 1. Kaplan-Meier overall survival curves of subgroups divided by hematological markers in triple-positive, *IDH* and *TERT* mutations, and *IDH* mutation only molecular groups of lower-grade gliomas. (**A**–**D**) In triple-positive group of lower-grade gliomas, the OS of patients with NLR>2.00 or PLR>166.15 or MLR>0.33 or AGR>1.78does not significantly differ from that of patients with NLR≤2.00 or MLR≤0.29or MLR≤0.33 or AGR≤1.78 (NLR P=0.257, PLR P=0.270, MLR P=0.497, AGR P=0.885). (**E**, **F**) In *IDH* and *TERT* mutations group of lower-grade gliomas, the OS of patients with PLR>166.00 or AGR>1.76 does not significantly differ from that of patients with PLR≤166.00 or AGR≤1.76 (PLR P=0.599, AGR P=0.493). (**G**, **H**) In *IDH* mutation only group of lower-grade gliomas, the OS of patients with MLR>0.33 or AGR>1.89 does not significantly differ from that of patients with MLR≤0.33 or AGR≤1.89 (MLR P=0.776, AGR P=0.251).

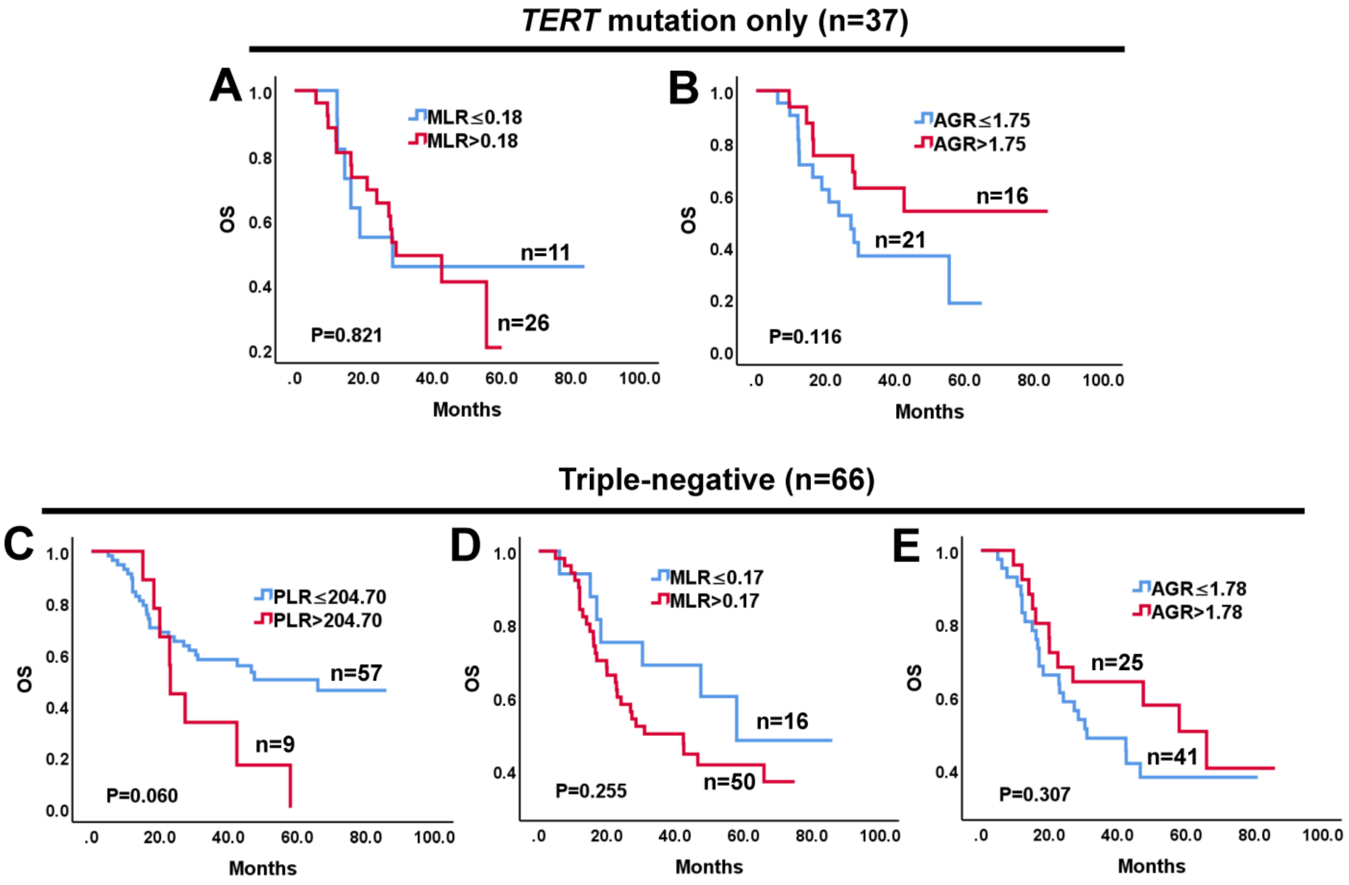

Supplementary Figure 2. Kaplan-Meier overall survival curves of subgroups divided by hematological markers in *TERT* mutation only, and triple-negative groups of lower-grade gliomas. (**A**–**B**) In *TERT* mutation only group of lower-grade gliomas, the OS of patients with MLR>0.18 or AGR>1.75 does not significantly differ from that of patients with MLR≤0.18 or AGR≤1.75 (MLR P=0.821, AGR P=0.116). (**C**–**E**) In triple-negative group of lower-grade gliomas, the OS of patients with PLR>204.70 or MLR>0.17 or AGR>1.78 does not significantly differ from that of patients with PLR≤204.70 or MLR≤0.17 or AGR≤1.78 (PLR P=0.060, MLR P=0.255, AGR P=0.307).
