# Supplementary material for: Prognostic value of preoperative hematological markers combined with molecular pathology in patients with diffuse gliomas
Source: Aging (Albany NY). 2019 Aug 23;11(16):6252–72. doi: 10.18632/aging.102186 (PMC6738441; doi:10.18632/aging.102186)
Supplement: Supplementary Tables [file aging-11-102186-s002.pdf]

SUPPLEMENTARY TABLES

Supplementary Table 1. Clinico-pathological features of the cohort (n=592).

Baseline characteristics	Number
Gender	
Male	335 (56.59%)
Female	257 (43.41%)
Age	
Mean ±SD	48.26±1.503
Median (range)	42 (16-82)
Extent of resection	
Complete	456 (77.03%)
Incomplete	136 (22.97%)
RT	
Yes	459 (77.53%)
No	133 (22.47%)
CHT	
TMZ	186 (31.42%)
NMST/FMST	156 (26.35%)
No	250 (42.23%)
Tumor grade	
II	282 (47.64%)
III	122 (20.61%)
IV	188 (31.76%)
IDH mutations	
IDH1 mutation	235 (41.01%)
R132C	2 (0.35%)
R132G	1 (0.17%)
R132H	227 (39.62%)
R132S	2 (0.35%)
R133H	1 (0.17%)
R134H	1 (0.17%)
R135H	1 (0.17%)
IDH2 mutation	11 (1.92%)
R132S	1 (0.17%)
R172G	1 (0.17%)
R172K	5 (0.87%)
R172W	4 (0.70%)
IDH wild type	327 (57.07%)
TERT promoter mutations	

TERT promoter mutation	286 (49.91%)
C228T	212 (37.00%)
C250T	74 (12.91%)
TERT promoter wild type	287 (50.09%)
1p/19q deletion	
Only 1p deletion	15 (2.62%)
Only 19q deletion	35 (6.11%)
1p/19q codeletion	139 (24.26%)
1p/19q intact	384 (67.02%)
Hematological marker (n=528) [a]	
NLR (Mean ±SD)	3.02±2.81
PLR (Mean ±SD)	129.28±62.17
MLR (Mean ±SD)	0.28±0.13
AGR (Mean ±SD)	1.77±0.36
MPV (Mean ±SD)	8.46±0.05
PDW (Mean ±SD)	16.47±0.02
Molecular group	
Grade II-IV (n=573) [b]	
Triple-positive	103 (17.98%)
IDH and *TERT* mutations	19 (3.32%)
IDH mutation only	108 (18.85%)
TERT mutation only	155 (27.05%)
Triple-negative	144 (25.13%)
Other	44 (7.7%)
Lower-grade gliomas (n=392)	
Triple-positive	103 (26.28%)
IDH and *TERT* mutations	19 (4.85%)
IDH mutation only	100 (25.51%)
TERT mutation only	48 (12.24%)
Triple-negative	78 (19.90%)
Other	44 (11.22%)
Grade IV glioma (n=181)	
IDH mutation only	8 (4.42%)
TERT mutation only	107 (59.12%)
Triple-negative	66 (36.46%)

[a]64 cases were excluded from the 592 cases due to conditions that could influence hematological makers
[b]19 cases were excluded from the 592 cases due to unavailability of FFPE tissues of the tumors.
RT: radiation therapy, indicating postoperative radiation therapy after first operation
CHT: chemotherapy, indicating postoperative chemotherapy after first operation
TMZ: temozolomide, FMST: fotemustine, NMST: nimustine

Supplementary Table 2. Univariate analysis of prognostic factors for OS in lower-grade gliomas (n=404).

Factors	No. of cases	5-year OS (%)	P-value
Sex			
Male	228	59.6	P=0.276
Female	176	66.8	
Age			
≤40	146	73.7	**P<0.001**
>40	258	56.1	
KPS			
≤80	149	56.7	**P=0.019**
>80	255	66.7	
Extent of resection			
Gross total	326	65.2	**P=0.004**
Subtotal	78	49.9	
RT			
Yes	324	65.9	**P=0.019**
No	80	48.1	
CHT			
Yes	313	64.7	P=0.129
No	91	54.4	
Grade			
II	282	76.3	**P<0.001**
III	122	27.8	
Molecular group(n=348) [a, b]			
Triple-positive	103	90.2	**P<0.001**
IDH and *TERT* mutations	19	67.4	
IDH mutation only	100	70.0	
TERT mutation only	48	24.0	
Triple-negative	78	38.3	

[a] 12 cases were excluded due to unavailability of FFPE tissues of the tumors, and 44 cases of other combinations of the three molecular markers were excluded.

[b] multiple comparisons for molecular groups are listed in Supplementary Table 3

OS: overall survival; KPS: Karnofsky Performance Status; RT: radiation therapy, indicating postoperative radiation therapy after first operation; CHT: chemotherapy, indicating postoperative chemotherapy after first operation

Supplementary Table 3. The classification of radiotherapy, chemotherapy and chemotherapy program in WHO II-III gliomas (n=403).

	Astrocytoma (%)	Oligodendroglioma or Oligoastrocytomas (%)
Only RT	14 (9.03)	31 (12.5)
Only CHT	10 (6.45)	24 (9.68)
RT and CHT	113 (72.90)	166 (66.94)
No RT nor CHT	18 (11.61)	27 (10.89)
CHT program		
TMZ	36 (29.27)	68 (35.79)
FMST/NMST	49 (39.84)	81 (42.63)
NA	38 (30.89)	41 (21.58)

RT: radiotherapy; CHT: chemotherapy; TMZ: temozolomide; FMST: fotemustine; NMST: nimustine; NA: not available

Supplementary Table 4. Univariate analysis of molecular groups of lower-grade gliomas with multiple comparisons (n=348[a]).

Molecular group 1 vs Molecular group 2	No. of cases	5-year OS (%)	P-value[b]
Triple-positive vs *IDH* and *TERT* mutations	103 vs 19	90.2 vs 67.4	P=0.096
Triple-positive vs *IDH* mutation only	103 vs 100	90.2 vs 70.0	P=0.009
Triple-positive vs *TERT* mutation only	103 vs 48	90.2 vs 24.0	**P<0.001**
Triple-positive vs Triple negative	103 vs 78	90.2 vs 38.3	**P<0.001**
IDH and *TERT* mutations vs *IDH* mutation only	19 vs 100	67.4 vs 70.1	P=0.994
IDH and *TERT* mutations vs *TERT* mutation only	19 vs 48	67.4 vs 24.0	**P=0.002**
IDH and *TERT* mutations vs Triple negative	19 vs 78	67.4 vs 38.3	P=0.011
IDH mutation only vs *TERT* mutation only	100 vs 48	70.0 vs 24.0	**P<0.001**
IDH mutation only vs Triple negative	100 vs 78	70.0 vs 38.3	**P<0.001**
TERT mutation only vs Triple negative	48 vs 78	24.0 vs 38.3	P=0.162

[a]12 cases were excluded due to unavailability of FFPE tissues of the tumors, and 44 cases of other combinations of the three molecular markers were excluded.
[b]To correct for multiple comparisons, a Bonferroni adjusted P value of 0.05/10(number of times of comparisons) =0.005 was adopted as the significance threshold

Supplementary Table 5. Univariate analysis of prognostic factors for OS in Grade IV glioma (n=188).

Factors	No. of cases	5-year OS (%)	P-value
Sex			
Male	107	4.8	P=0.488
Female	81	2.2	
Age			
≤62	50	3.5	**P=0.037**
>62	138	4.0	
KPS			
≤80	45	9.7	P=0.151
>80	143	2.6	
Extent of resection			
Gross total	130	4.4	**P<0.001**
Subtotal	58	1.9	
RT			
Yes	135	6.6	**P=0.006**
No	53	6.3	
CHT			
Yes	125	0.0	**P<0.001**
No	63	3.2	
Molecular group (n=181)[a]			
IDH mutation only	8	25.0	P=0.285
TERT mutation only	107	2.7	
Triple-negative	66	3.7	

[a]7 cases were excluded due to unavailability of FFPE tissues of the tumors.
OS: overall survival; KPS: Karnofsky Performance Status; RT: radiation therapy, indicating postoperative radiation therapy after first operation CHT: chemotherapy, indicating postoperative chemotherapy after first operation

Supplementary Table 6. P-value in the univariate analysis of subgroups of lower-grade gliomas with multiple comparisons (n=348[a]).

Subgroup 1 vs Subgroup 2	P-value[b]
Triple positive vs IDH and TERT mutation-Low NLR	**P<0.001**
Triple positive vs IDH mutation only-Low NLR	**P<0.001**
Triple positive vs TERT mutation only-High NLR	**P<0.001**
Triple positive vs TERT mutation only-Low NLR	**P<0.001**
Triple positive vs Triple-negative-Low NLR	**P<0.001**
IDH and TERT mutation-High NLR vs TERT mutation only-Low NLR	**P<0.001**
IDH and TERT mutation-High NLR vs Triple-negative-Low NLR	**P=0.001**
IDH and TERT mutation-Low NLR vs Triple-negative-Low NLR	**P<0.001**
IDH mutation only-High NLR vs TERT mutation only-High NLR	**P<0.001**
IDH mutation only-High NLR vs TERT mutation only-Low NLR	**P<0.001**
IDH mutation only-High NLR vs Triple-negative-Low NLR	**P<0.001**
IDH mutation only-Low NLR vs TERT mutation only-Low NLR	**P<0.001**
IDH mutation only-Low NLR vs Triple-negative-Low NLR	**P=0.001**
TERT mutation only-High NLR vs TERT mutation only-Low NLR	**P<0.001**
TERT mutation only-Low NLR vs Triple-negative-High NLR	**P<0.001**
TERT mutation only-Low NLR vs Triple-negative-Low NLR	**P<0.001**
Triple-negative-High NLR vs Triple-negative-Low NLR	**P=0.001**

[a]12 cases were excluded due to unavailability of FFPE tissues of the tumors, and 44 cases of other combinations of the three molecular markers were excluded.
[b]To correct for multiple comparisons, a Bonferroni adjusted P value of 0.05/36(number of times of comparisons) =0.0014 was adopted as the significance threshold
We removed the date that P>0.05 for a more streamlined form.

Supplementary Table 7. Univariate analysis of risk group of lower-grade gliomas with multiple comparisons (n=348[a]).

Risk group 1 vs Risk group 2	No.of cases	5-year OS (%)	P-value[b]
Low risk vs Intermediate-I	179 vs 98	85.5 vs 53.0	**P<0.001**
Low risk vs High risk	179 vs 18	85.5 vs 0.0	**P<0.001**
Low risk vs Intermediate-II	179 vs 53	85.5 vs 23.4	**P<0.001**
Intermediate-I vs High risk	98 vs 18	53.0 vs 0.0	**P<0.001**
Intermediate-I vs Intermediate-II	98 vs 53	53.0 vs 23.4	**P<0.001**
High risk vs Intermediate-II	18 vs 53	0.0 vs 23.4	**P<0.001**

[a]12 cases were excluded due to unavailability of FFPE tissues of the tumors, and 44 cases of other combinations of the three molecular markers were excluded.
[b]To correct for multiple comparisons, a Bonferroni adjusted P value of 0.05/6(number of times of comparisons) =0.0083 was adopted as the significance threshold
